# Study on the Expression Level of CAVIN3 Gene and the Prognosis of Radiotherapy in Patients with Cervical Cancer

**DOI:** 10.3390/cancers18162551

**Published:** 2026-08-08

**Authors:** Ying Ye, Zhichao Fu, Xinpeng Wang, Shilong Deng, Lvjuan Cai, Jing Feng, Fengmei Wang

**Affiliations:** 1 Department of Radiotherapy, Fuzong Clinical Medical College of Fujian Medical University (900th Hospital), Fuzhou 350025, China; 2 Department of Obstetrics and Gynecology, Fuzong Clinical Medical College of Fujian Medical University (900th Hospital), Fuzhou 350025, China

**Keywords:** cervical cancer, gynecological cancer, CAVIN3, prognosis, survival

## Abstract

Objective: This study aims to investigate the expression of CAVIN3 in cervical cancer, its effect on radiotherapy outcomes, and its potential molecular mechanisms. Materials and Methods: The GEPIA2 database was used to screen genes that may affect cervical cancer prognosis. Then, we analyzed 133 cervical cancer patients from the TCGA database who received radiotherapy to evaluate CAVIN3 gene expression and function. Receiver operating characteristic (ROC) curves and Cox regression models were employed to assess the diagnostic value of CAVIN3 and its association with radiotherapy outcomes. GSEA, GO, and KEGG databases were used to perform pathway enrichment analysis of CAVIN3-related signaling pathways. We also collected pretreatment biopsy specimens from 66 patients who were subsequently treated with radiotherapy from our center to validate the findings from the database analysis. Results: In the TCGA database, CAVIN3 expression was significantly lower in cervical cancer tissues than in normal cervical tissues (*p* = 0.019), a finding that was independently corroborated in our own cohort (*p* = 0.002). Receiver operating characteristic analysis yielded area-under-the-curve values of 0.894 for the TCGA RNA-seq data and 0.947 for our center, underscoring the gene’s potential diagnostic utility. Next, we stratified cervical cancer patients who received radiotherapy by intratumoral CAVIN3 levels. Notably, the low-expression group consistently showed better treatment outcomes. In the TCGA cohort, high CAVIN3 expression was associated with significantly shorter median overall survival (mOS, 31.8 months versus not reached within follow-up; *p* = 0.043). This pattern was confirmed in our validation cohort, where high CAVIN3 expression predicted markedly inferior median progression-free survival (mPFS, 9.8 vs. 59.9 months; *p* < 0.05) and mOS (17.45 months vs. not reached; *p* < 0.05). Consistent with these survival differences, the objective response rate to radiotherapy was lower in the high-expression group than in the low-expression group (56.5% vs. 88.2%, *p* = 0.006), revealing better radiotherapy response among tumors with low CAVIN3 expression. Enrichment analysis revealed that the high CAVIN3 expression group was primarily enriched in pathways related to extracellular matrix formation and remodeling, extracellular matrix–cell membrane interactions, cell adhesion and migration, integrin β1 signaling, and the regulation of cell growth, differentiation, and apoptosis. Together, these functions indicate a more differentiated, matrix-attached phenotype that can promote radioresistance. Conversely, low CAVIN3 expression likely reflects a poorly differentiated state with diminished matrix interaction, which renders the tumor more vulnerable to radiation. Conclusions: CAVIN3 downregulation is a frequent event in cervical cancer and may contribute to tumor susceptibility. Paradoxically, within tumors, low expression of CAVIN3 is associated with an enhanced response to radiotherapy, likely stemming from the underlying phenotype characterized by poor differentiation and heightened radiosensitivity. Therefore, CAVIN3 expression levels are correlated with cervical cancer development and the radiotherapy response of cervical cancer patients.

## Simple Summary

CAVIN3 is downregulated in cervical cancer and holds promise as a potential diagnostic biomarker. This study investigated CAVIN3 expression and its impact on radiotherapy outcomes utilizing TCGA data (*n* = 133) and an independent validation cohort (*n* = 66). Low CAVIN3 expression was associated with a significantly improved response to radiotherapy, with a higher objective response rate (88.2% vs. 56.5%) and longer progression-free and overall survival times. High CAVIN3 expression may be associated with extracellular matrix-related pathways and a more differentiated, radioresistant phenotype. Thus, CAVIN3 expression levels correlate with cervical cancer development and the radiotherapy response of cervical cancer patients.

## 1. Introduction

Statistics on the prevalence of malignant tumors in China in 2022 show that cervical cancer ranks fifth in incidence and sixth in mortality. In 2022, there were 150,700 new cases of cervical cancer nationwide, accounting for an incidence rate of 21.18 per 100,000 among female malignancies, with 55,700 deaths, accounting for a mortality rate of 8.06 per 100,000 [1]. According to 2022 GLOBOCAN (Global Cancer Observatory) data [2], approximately 600,000 new cases of cervical cancer were reported globally. Although vaccination rates have risen in some areas, cervical cancer remains one of the most common reproductive system cancers in women.

In the treatment of cervical cancer, concurrent chemoradiotherapy is the standard treatment for locally advanced cervical cancer that cannot be surgically removed, yet its overall effectiveness is still limited. Therefore, there is a pressing need to find biomarkers that predict cervical cancer radiotherapy outcomes to guide clinical treatment decisions for patients.

Caveolae are small, flask-shaped invaginations of the plasma membrane [3], consisting mainly of the caveolin and cavin protein families. So far, seven members of the Caveolae family have been discovered: CAV1, CAV2, CAV3, CAVIN1, CAVIN2, CAVIN3, and CAVIN4. Caveolae proteins often play a paradoxical role in tumor development, as they are associated with both promoting tumor progression and suppressing tumor growth [4,5].

The CAVIN3 (caveolae-associated protein 3) gene encodes a 261-amino acid protein known as PRKCDBP (protein kinase C delta-binding protein), which contains a leucine zipper, a protein kinase C (PKC)-binding site, a PKC phosphorylation site, a phosphatidylserine-binding site, and two PEST domains [6]. By binding to caveolin, it plays a key role in forming and stabilizing caveolae, promoting their assembly and maintenance [7]. Studies indicate that CAVIN3 expression varies across cell types, with particularly high levels in adipose tissue; these levels are closely linked to its role in adipocyte differentiation [8]. Caveolae not only contribute to cell membrane stability but also are involved in intracellular signaling and cargo transport. The release of CAVIN3 coincides with caveolae disassembly, a process critical for cellular stress responses. Under mechanical stress or UV radiation, caveolae disassemble, releasing CAVIN3 to interact with other intracellular proteins and regulate cellular metabolism and stress responses. These findings reveal that CAVIN3 is not only a structural component of caveolae but also plays a vital role in cell membrane dynamics.

CAVIN3’s role in cell proliferation and apoptosis has garnered significant attention. CAVIN3 absence heightens cell sensitivity to stressful stimuli such as UV radiation, thereby promoting apoptosis. This process hinges on CAVIN3’s interaction with BRCA1, through which it modulates DNA repair capacity and apoptotic signaling pathways. Additionally, CAVIN3 has been confirmed to regulate cell proliferation. Studies show that its overexpression promotes proliferation, while downregulation inhibits growth [8]. This effect may stem from CAVIN3’s involvement in signaling pathways, particularly in the tumor microenvironment, where altered CAVIN3 expression may directly promote or suppress tumor cell growth and survival.

CAVIN3 plays a key role in cell signaling regulation. Studies show that CAVIN3 regulates cell survival signals by interacting with various receptor tyrosine kinases (RTKs). For instance, CAVIN3—ROR1 interaction is crucial for maintaining cell survival signals through AKT pathway activation [9]. In tumor cells, CAVIN3 expression levels often correlate with tumor aggressiveness. Research indicates that CAVIN3 shows downregulation in multiple cancer cell lines, indicating possible tumor suppressor activity [10]. CAVIN3 deficiency decreases cells’ sensitivity to external signals, promoting tumor cell survival and proliferation. This suggests that CAVIN3 could be a potential therapeutic target. Since CAVIN3 deficiency promotes tumor cell survival and proliferation, targeting its expression may restore normal cell-signaling balance, thereby improving treatment outcomes.

TME (tumor microenvironment) refers to the complex local ecosystem surrounding tumor cells. It is composed of tumor cells, stromal cells (fibroblasts, immune cells, endothelial cells), extracellular matrix, signaling molecules (cytokines, growth factors), and chemical conditions (hypoxia, acidity). CAVIN3, a key membrane protein, plays multiple roles in the tumor microenvironment, influencing tumor cell biology and affecting tumor development and outcomes by regulating tumor-associated cells. TNFα, a multifunctional cytokine, plays significant roles in inflammation, angiogenesis, tissue remodeling, and tumor growth [11]. PRKCDBP, which promotes apoptosis and suppresses tumors, is often silenced by promoter methylation in colorectal cancer. When stimulated by TNFα, NF-κB directly activates the transcription of PRKCDBP. Thus, PRKCDBP loss may drive tumor progression by reducing cell sensitivity to TNFα and other stresses, particularly in chronic inflammatory microenvironments [6].

In conclusion, CAVIN3 affects tumor-associated macrophages, extracellular matrix, and immune evasion through various mechanisms in the tumor microenvironment, influencing tumor development. These findings provide new perspectives on CAVIN3’s therapeutic potential. In particular, our study reveals CAVIN3’s importance in cervical cancer diagnosis and radiation response, which further validates it as a valuable clinical biomarker. CAVIN3 can serve as a potential clinical biomarker as its expression levels in cervical cancer tissues may be used to predict the prognosis of patients and their response to radiotherapy, which is determined by analyzing the correlation between CAVIN3 expression and tumor cell radiosensitivity and patient survival rates.

## 2. Materials and Methods

### 2.1. Gene Screening

This study conducted a systematic evaluation of the CAVIN3 gene’s expression characteristics across pan-cancer tissues by using the differential gene expression analysis module of the GEPIA2 (Gene Expression Profiling Interactive Analysis) database. We used the “Differential Genes” function in the “Expression Analysis” module with screening criteria of q-value < 0.05 and |log2FC| > 1, and then employed ANOVA variance analysis and the LIMMA algorithm for two-step validation. The analysis included 33 tumor types from the TCGA database, such as CESC (Cervical Squamous Cell Carcinoma and Endocervical Adenocarcinoma), along with paired adjacent normal tissue samples. The results showed that CAVIN3 was significantly downregulated in 13 malignancies, including CESC. Additionally, the survival package was used to perform a proportional hazards assumption test for CAVIN3’s survival differences across pan-cancer, followed by a Cox regression analysis to assess its impact on survival in various tumors. Forest plot visualization was generated using ggplot2 (v3.4.4).

### 2.2. Data Collection and Processing

This study obtained multi-omics datasets for cervical cancer from the TCGA database (http://cancergenome.nih.gov/ (accessed on 10 November 2023)). Raw mRNA-seq data (FPKM format) from 306 cervical cancer tissues and 3 adjacent normal tissues were downloaded via the GDC Data Portal and normalized using the edgeR package (version 3.38.1).

#### 2.2.1. Inclusion/Exclusion Criteria

##### TCGA Database Criteria

Inclusion criteria: (1) histologically confirmed primary cervical cancer; (2) underwent radiotherapy. Exclusion criteria: (1) secondary cervical malignancies; (2) concurrent or prior history of other malignancies; (3) had undergone radical hysterectomy; (4) patients lost to follow-up. Following rigorous screening, 133 cases with corresponding gene expression data were included. Among these 133 cases, 57 cases had imaging response assessments, and 43 cases with complete data were included in survival prognosis analysis.

##### Institutional Validation Cohort Criteria

Inclusion criteria: (1) Histologically confirmed primary cervical cancer; (2) received external beam radiotherapy as first-line treatment; (3) pathological specimens available from our center; (4) complete imaging data available. Exclusion criteria: (1) secondary cervical malignancies; (2) concurrent or prior history of other malignancies; (3) had undergone radical hysterectomy; (4) patients lost to follow-up. Following rigorous screening, 66 eligible cases with complete data were included for validation.

#### 2.2.2. Definition of Efficacy Evaluation Terms [12]

CR (Complete Response, also known as complete remission): All target lesions disappear, and the short axis of any pathological lymph nodes (whether target or non-target) must be reduced to less than 10 mm.

PR (Partial Response): The sum of diameters of target lesions decreases by at least 30% compared with the baseline sum.

PD (Progressive Disease): The sum of diameters of target lesions increases by at least 20% compared with the smallest sum recorded during the study (including the baseline sum if it is the smallest). In addition to the relative 20% increase, the sum must demonstrate an absolute increase of at least 5 mm. It should be noted that the appearance of one or more new lesions is classified as progression.

SD (Stable Disease): The tumor does not show shrinkage that meets PR criteria or increase that meets PD criteria, compared with the smallest sum of diameters during the study.

ORR (Objective Response Rate) = (CR + PR)/total number of cases × 100%.

OS (Overall Survival): Time from treatment initiation to death from any cause.

PFS (Progression-Free Survival): Time from treatment initiation to tumor progression (PD) or death from any cause, whichever occurs first.

Univariate and multivariate analyses were performed using the Cox proportional hazards model. The variables used in these analyses included age (stratified using thresholds based on median ages: 46 years from TCGA database and 56 years from this center), FIGO stage, tumor differentiation grade, body mass index (BMI), gene expression levels (grouped by X-tile-determined optimal cutoff values), histologic grade (G), etc. Survival curves were plotted using the Kaplan–Meier method, and group differences were assessed via log-rank test.

#### 2.2.3. Ethical Statement

This study used data from the open access TCGA database as well as clinical data collected from the 900th Hospital of the PLA Joint Logistics Support Force. All TCGA data were collected with patient informed consent. Our institution collected data from 66 patients who received primary radiotherapy between January 2017 and December 2022 and had pathological biopsy specimens obtained at our hospital prior to treatment. All methods were carried out in accordance with relevant guidelines and regulations, including the principles of the Declaration of Helsinki. This study was approved by the Ethics Committee of the 900th Hospital of the Joint Logistics Support Force (Ethics Review Section No. 2024-031).

### 2.3. Immunohistochemical Analysis of the Validation Cohort

#### 2.3.1. Sample Preparation and Quality Control

Our validation cohort comprised cases from our center, including 66 cervical cancer biopsy specimens and 3 matched histologically normal adjacent tissue samples collected from 2017 to 2022. Fresh biopsy specimens were fixed in 40 g/L neutral formaldehyde for 24 h, dehydrated through graded ethanol, and embedded in paraffin to prepare 4 μm continuous sections.

#### 2.3.2. Immunohistochemistry

We used the EliVision method, with results examined under a light microscope. The PRKCDBP Polyclonal Antibody was purchased from Proteintech Group (Chicago, IL, USA; Catalog No. 16250-1-AP). The non-biotin universal two-step immunohistochemistry kit (Mouse/Rabbit Enhanced Polymer Detection System) was obtained from Golden Bridge Biotechnology (Beijing, China). Parametrial tissue served as the positive control.

#### 2.3.3. Interpretation Criteria

**Figure 1:**
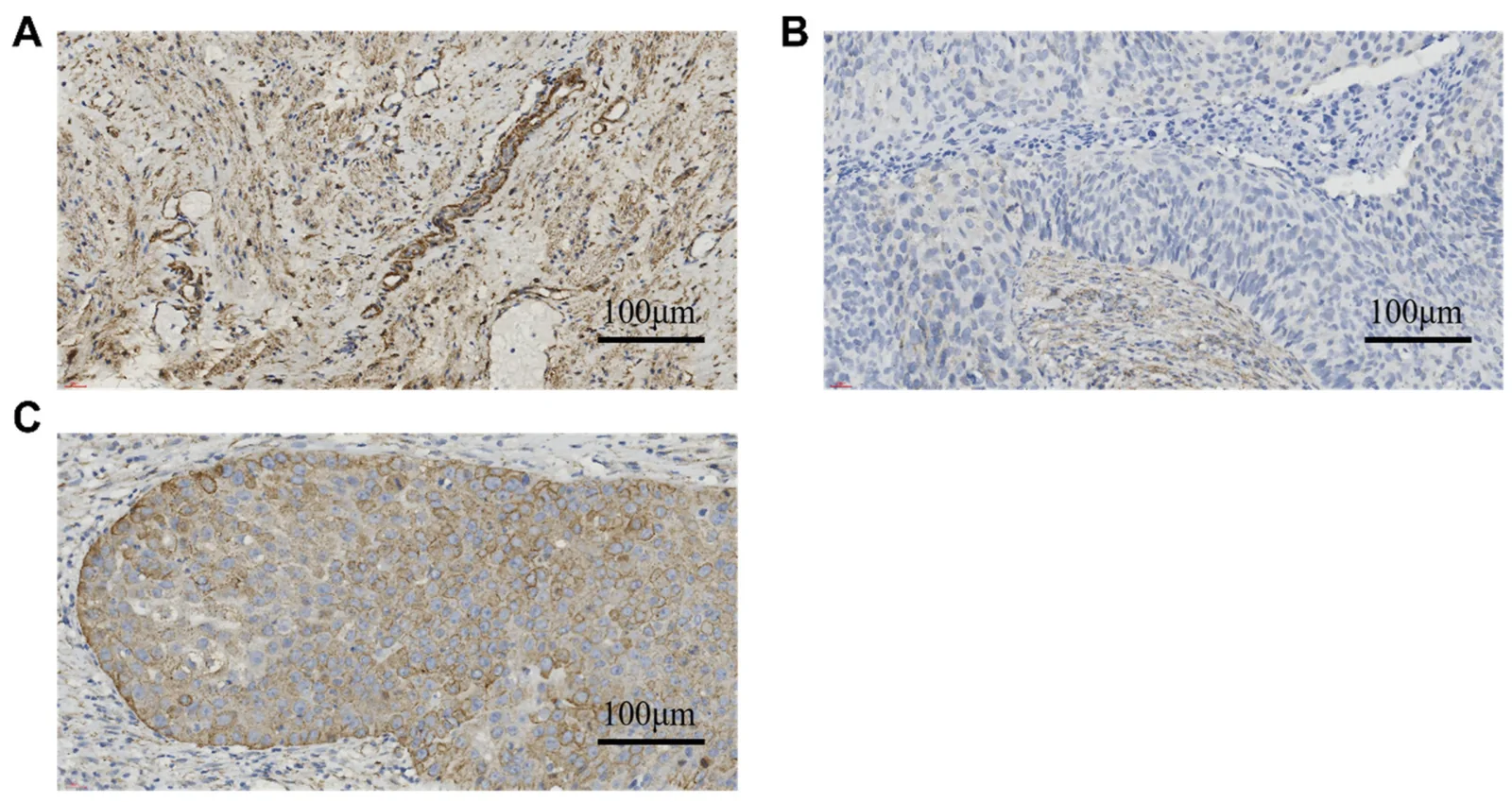
Pathological section CAVIN3 immunohistochemical staining image (magnification of 40×). (**A**) Positive control of normal tissue; (**B**) negative result of cervical tumor tissue; (**C**) positive result of cervical tumor tissue.

CAVIN3 positive expression appears as brownish-yellow particles in the cytoplasm and caveolae (small membrane invaginations) of the cell membrane (Figure 1). Cells with dark brown cytoplasm and membranes are classified as strongly stained; those with yellow or brown cytoplasm and membranes are defined as moderately stained; and those with pale yellow or faintly stained cytoplasm and membranes are considered weakly stained. No staining is observed in the nucleus. The expression of the CAVIN3 gene is quantified using the H-score, which is calculated as H-score = (P_1_ × 1) + (P_2_ × 2) + (P_3_ × 3), where P_1_, P_2_, and P_3_ are the percentages of weakly, moderately, and strongly positive cells, respectively (P_1_ + P_2_ + P_3_ = 100%). The H-score ranges from 0 to 300. The optimal cut-off value is determined by X-tile v3.6.1, dividing samples into high- and low-expression groups.

### 2.4. Bioinformatics Analysis of CAVIN3 Data

#### 2.4.1. Potential Signaling Pathway Analysis of CAVIN3

##### Gene Set Enrichment Analysis (GSEA)

This study employed the Hallmark gene set (H collection) from MSigDB v2023.2 for GSEA analysis to reveal CAVIN3’s biological mechanisms by comparing the transcriptomic profiles of high vs. low CAVIN3 expression groups. Using the log2(FPKM+1) normalized expression matrix, GSEA software (v4.3.2) calculated Enrichment Scores (ESs), with 1000 gene permutations to control false-discovery rates. The weighted enrichment statistic (weighted_p1.0) was applied, with gene sets filtered using the following thresholds: absolute NES > 1.5, FDR < 0.25, and nominal *p*-value < 0.05.

Key steps included:

(1) Gene ranking: Constructing a ranked list based on Spearman’s rho between gene expression and CAVIN3 (metric = Signal2Noise).
(2) Pathway activation: Positive NES values suggest gene set activation in high CAVIN3 expressers and negative values in low expressers.
(3) Core enriched genes: Leading-edge analysis identified genes contributing >50% to ESs.
(4) Multiple-hypothesis correction: FDR adjustment via Benjamini–Hochberg method.

##### Functional Enrichment Analysis

Gene Ontology (GO) and Kyoto Encyclopedia of Genes and Genomes (KEGG) [13] analyses were performed. RNA sequencing data processed with *DESeq2* yielded 1286 differentially expressed genes (DEGs) (*p* < 0.05, |log2FoldChange| > 1).

GO analysis used clusterProfiler with human-derived data (FDR < 0.05), organizing the results into the following categories: Biological Process (BP); Molecular Function (MF); Cellular Component (CC).

Results were visualized via bar plots and bubble charts for clarity.

KEGG pathway analysis employed clusterProfiler’s hypergeometric distribution test (*p* < 0.05). Enriched pathways were mapped, highlighting significant genes.

### 2.5. Statistical Analysis

Wilcoxon signed-rank tests assessed CAVIN3–clinicopathological correlations. Survival ROC analysis in R evaluated CAVIN3’s diagnostic value (AUC reflects accuracy). Kaplan–Meier analysis (Survival package (v3.3.1)) compared overall survival (OS) between expression groups. TCGA data underwent univariable Cox analysis incorporating BMI (reference 18.5–23.9 kg/m^2^ = 1; <18.5 = 2, >23.9 = 3); hormone history (prior use = 1, never = 0); CAVIN3 expression (low = L; high = H); parity (≥3 = 1, <3 = 0); histology (squamous = 1, adenocarcinoma = 2); age (median 46 years; >46 = 2, ≤46 = 1); histological grade, HPV status (positive = 1), clinical stage. Multivariable Cox analysis determined CAVIN3’s independence as an OS risk factor. Institutional data incorporated tumor size (<4.8 cm = 0, ≥4.8 cm = 1); clinical stage, nodal status (metastasis = 1, none = 0); age (median 56 years; >56 = 2, ≤56 = 1); CAVIN3 grouping. All patients underwent primary radiotherapy, with first progression as the endpoint (PFS). Kaplan–Meier analysis compared the treatment response between expression groups (*p* < 0.05 significant). Data analysis used R (v4.2.1) and visualization used Adobe Photoshop CC.

### 2.6. Research Roadmap for TCGA Database Data Analysis

**Figure 2:**
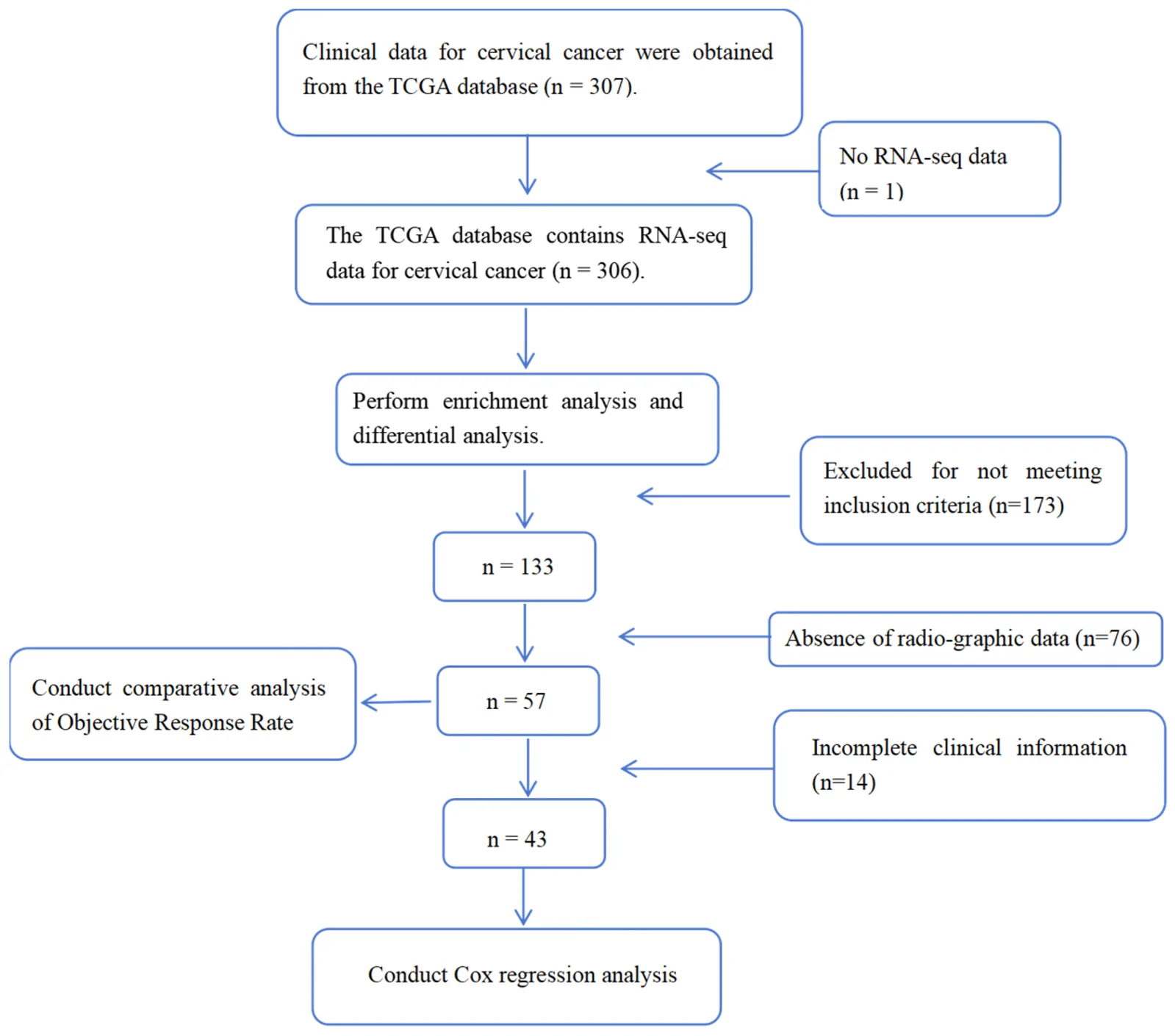
Research roadmap for data screening and analysis based on the TCGA database.

The TCGA transcriptomic dataset was systematically screened according to predefined inclusion and exclusion criteria. The overall data acquisition, filtering, and analytical workflow are summarized in the research roadmap shown in Figure 2.

## 3. Results

### 3.1. Patient Baseline Characteristics and Low Expression of CAVIN3 in Cervical Cancer Tissues

**Figure 3:**
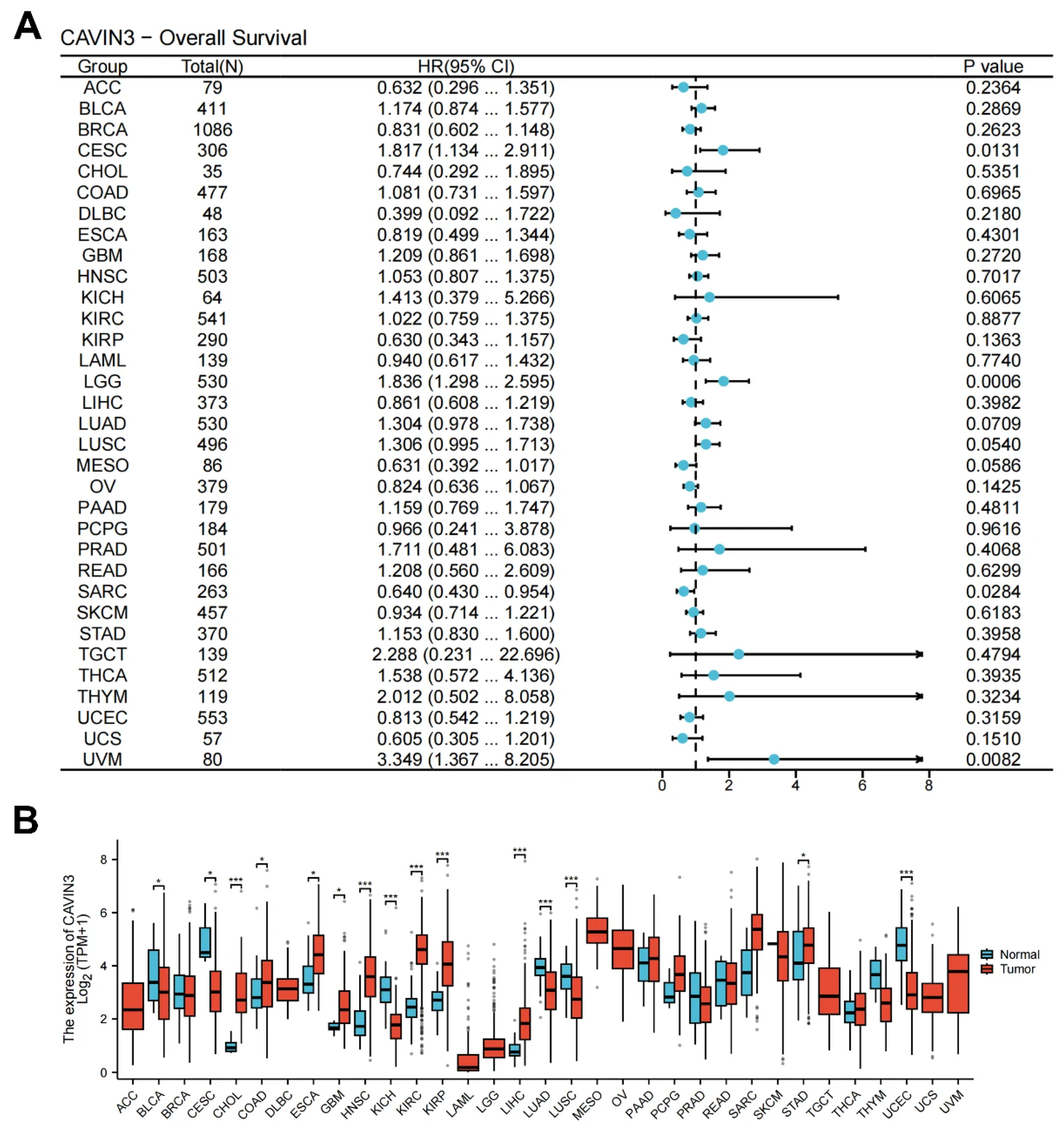
(**A**) Forest plot illustrating the survival outcomes associated with the CAVIN3 gene across 33 different tumor types. (**B**) Differences in the expression of the CAVIN3 gene across 33 types of tumors and adjacent tissues. * *p* < 0.05; *** *p* < 0.001.

To determine CAVIN3 expression across various tumors, we conducted a comprehensive analysis of 33 cancer types using the GEPIA database. The results showed that CAVIN3 expression was higher in tumor tissues than in adjacent normal tissues in seven cancer types: DLBC, ESCA, HNSC, KIRC, KIRP, PAAD, and THYM. Conversely, CAVIN3 expression was significantly lower in tumor tissues than in adjacent normal tissues in 13 cancer types: CESC, BLCA, BRCA, COAD, KICH, LUAD, LUSC, PRAD, READ, TGCT, THCA, UCEC, and UCS. Furthermore, pan-cancer survival analysis revealed that CAVIN3 had prognostic significance in CESC (Figure 3).

**Figure 4:**
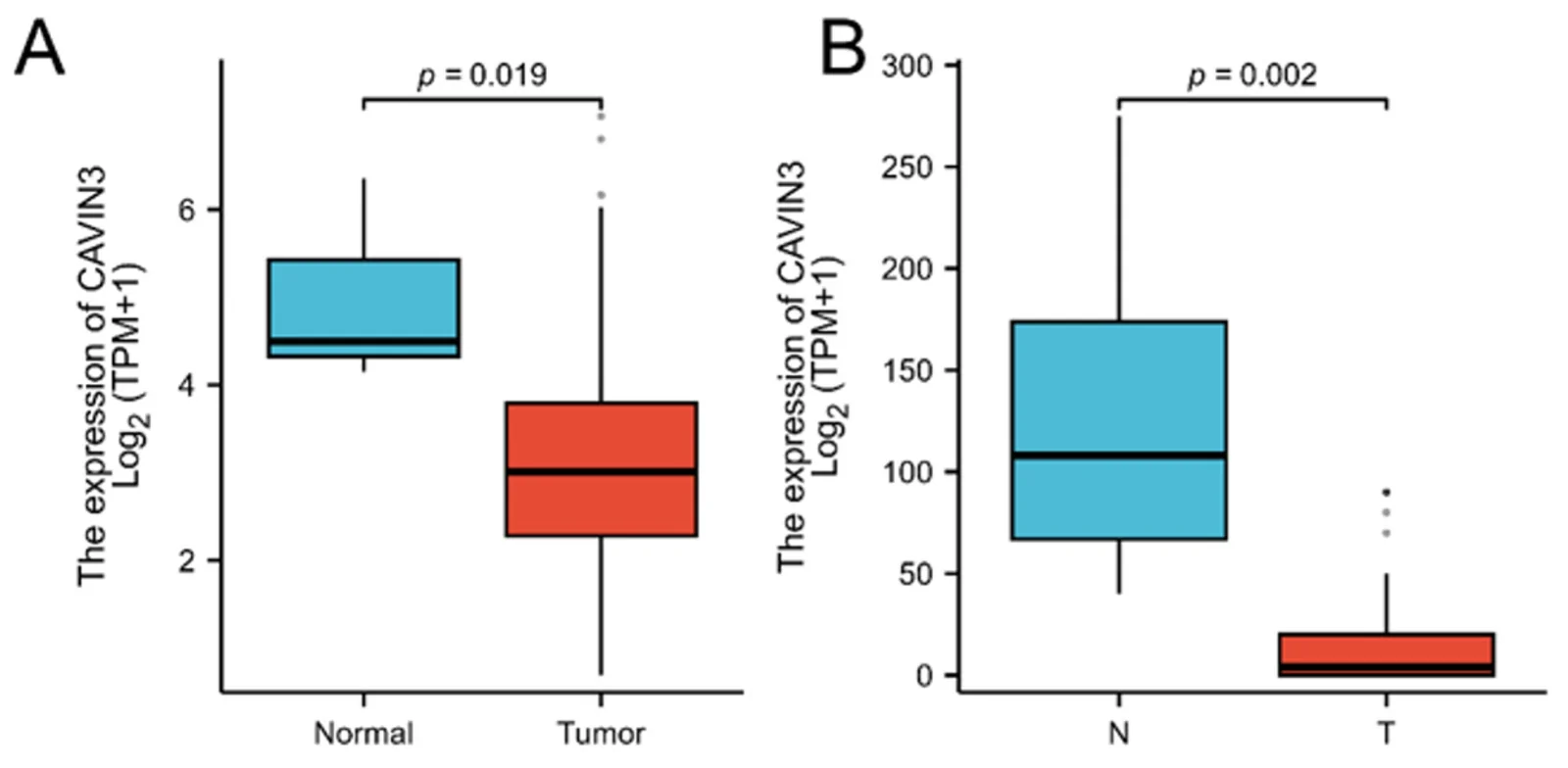
(**A**) CAVIN3 expression differences between cervical cancer tissues and adjacent normal tissues in the TCGA database. (**B**) CAVIN3 expression differences between cervical cancer tissues and adjacent normal tissues in our center.

To further verify CAVIN3 expression in cervical cancer, we analyzed 43 TCGA database cases meeting inclusion/exclusion criteria and 66 validation cases from our center, all with ECOG scores < 2. In our center’s cohort, based on the 2018 FIGO cervical cancer staging system, there were 19 stage II cases (28.8%), 36 stage III cases (54.5%), and 11 stage IV cases (16.7%). Histological analysis revealed 57 cases of squamous cell carcinoma (86.4%) and nine cases of adenocarcinoma (13.6%). All patients enrolled at our center demonstrated normal sinus rhythm on electrocardiography (ECG) (Table 1). Moreover, results also showed that CAVIN3 gene expression was significantly lower in cervical tumor tissues than in normal tissues (TCGA database: *p* = 0.019, our center *p* = 0.002) (Figure 4).

### 3.2. High CAVIN3 Expression Is an Independent Risk Factor for Cervical Cancer Prognosis

Among 307 cervical cancer cases in the TCGA database, we identified 43 cases with mRNA-seq data, radiotherapy history, and complete treatment response records. Univariate and multivariate Cox regression analyses assessed the independent prognostic value of CAVIN3 expression in cervical cancer patients undergoing radiotherapy. CAVIN3 showed low expression in cervical cancer tissues. Using X-tile software, we determined the optimal cutoff to divide the tissues into high-expression (Group H) and low-expression (Group L) groups. Cox analysis showed patients in the high-expression group had significantly shorter mOS than the low-expression group (31.8 months vs. median not reached, *p* = 0.043). Multivariate analysis confirmed high CAVIN3 expression and stage IV as independent risk factors for cervical cancer prognosis (Table 2).

**Figure 5:**
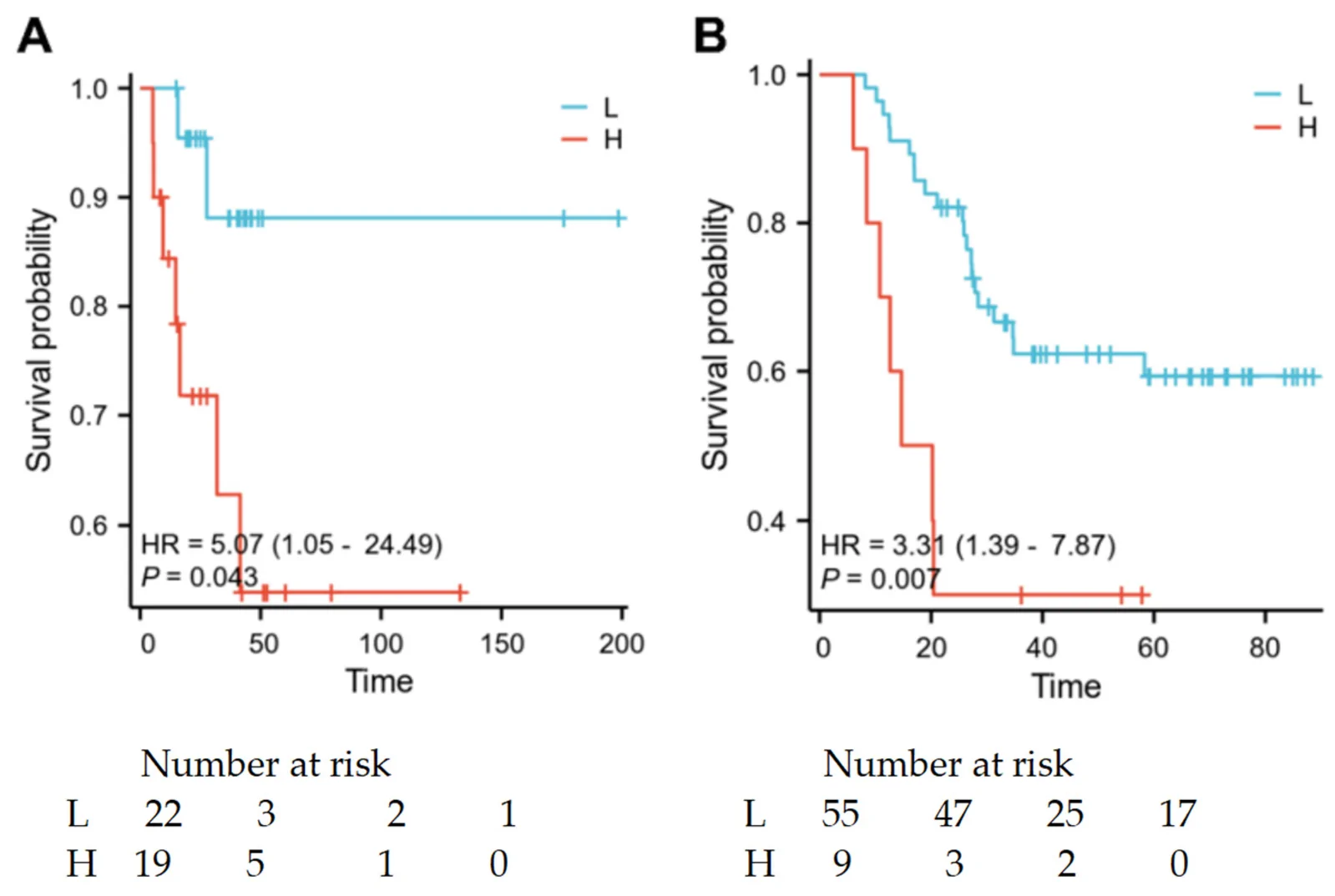
(**A**) Kaplan–Meier survival analysis stratified by CAVIN3 expression in TCGA. (**B**) Kaplan–Meier survival analysis stratified by CAVIN3 expression in our center.

Based on these results, and to further validate the findings, we analyzed data from our center’s cervical cancer patients, similarly demonstrating that high CAVIN3 expression is an independent prognostic risk factor. High-expression patients had significantly shorter mOS than low-expression patients (17.45 months vs. median not reached, *p* < 0.05). Multivariate analysis confirmed baseline tumor size ≥ 4.8 cm indicates poor prognosis (*p* < 0.05; Table 3, Figure 5).

### 3.3. Low CAVIN3 Expression Is Associated with an Improved Response to Radiotherapy in Cervical Cancer

Based on the analysis of the 57 cases with response assessment, the objective response rate (ORR) in the CAVIN3 high-expression group was 56.5% (13/23, 95% CI: 34.5–76.8%), while that in the CAVIN3 low-expression group was 88.2% (30/34, 95% CI: 72.6–96.7%). The absolute risk difference between the two groups was 31.7% (95% CI: 11.7–52.3%), which was statistically significant (χ^2^ = 7.46, *p* = 0.006). Logistic regression analysis, with the low-expression group as the reference, revealed that high CAVIN3 expression was associated with a significantly lower odds of achieving ORR (odds ratio [OR] = 0.173, 95% CI: 0.046–0.654, *p* = 0.010), corresponding to a 5.78-fold greater odds of response in the low-expression group relative to the high-expression group (95% CI: 1.53–21.7). These findings indicate that low CAVIN3 expression is significantly associated with a higher treatment response rate, and high CAVIN3 expression correlates with inferior therapeutic efficacy (Table 4).

**Figure 6:**
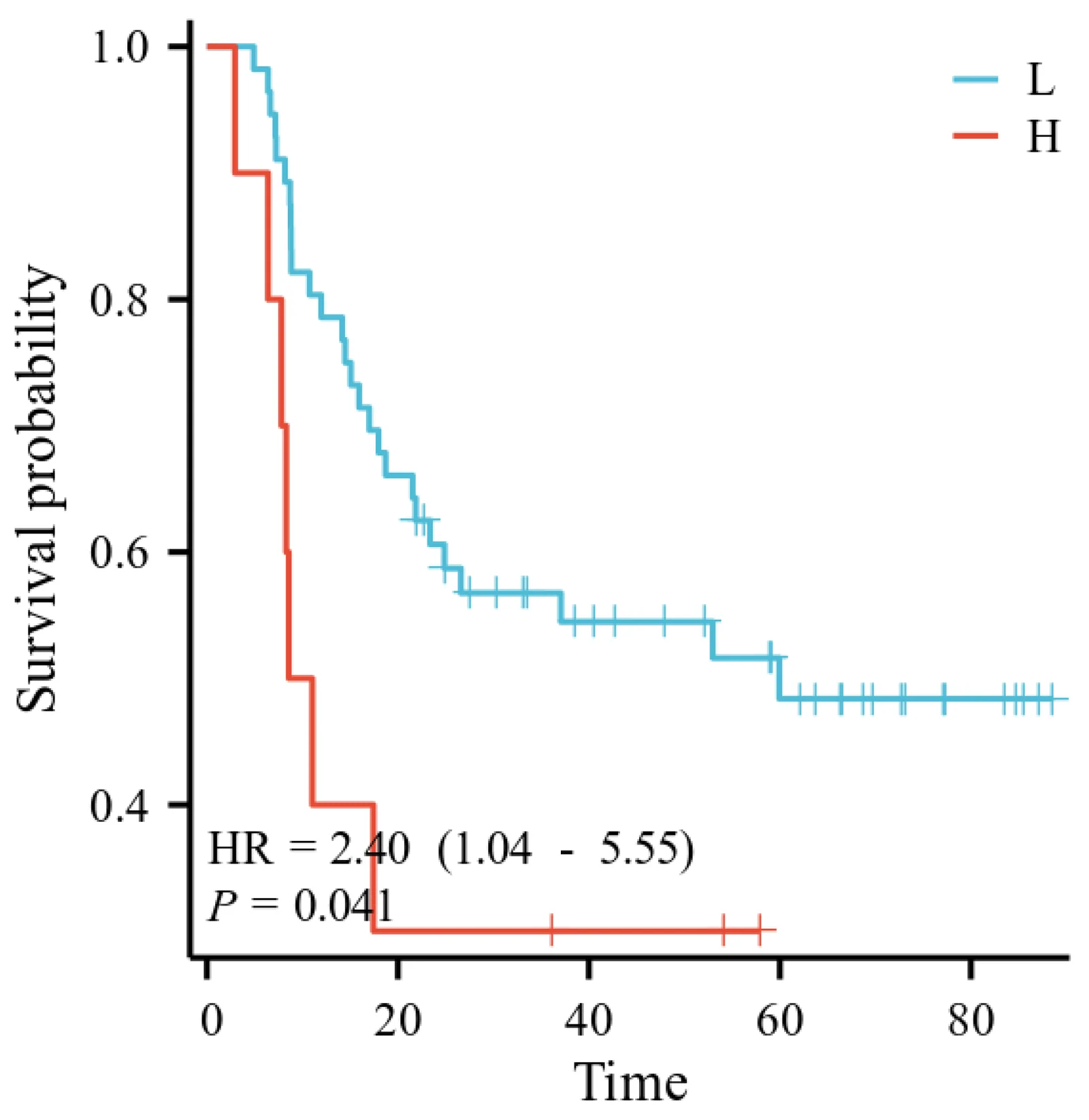
KM curves for PFS stratified by CAVIN3 expression levels (high vs. low) in our institutional validation cohort.

To further confirm these results, we validated these findings using clinical data from our center. All patients received first-line radiotherapy, with progression-free survival (PFS) as the primary endpoint. Cox multivariate analysis showed significantly worse PFS in the high-expression group post-radiotherapy (9.8 vs. 59.9 months, *p* < 0.05; Table 5, Figure 6). High CAVIN3 expression independently predicted poorer radiation outcomes.

### 3.4. Exploratory Evaluation of CAVIN3 Gene Expression in Cervical Cancer Versus Normal Tissue

**Figure 7:**
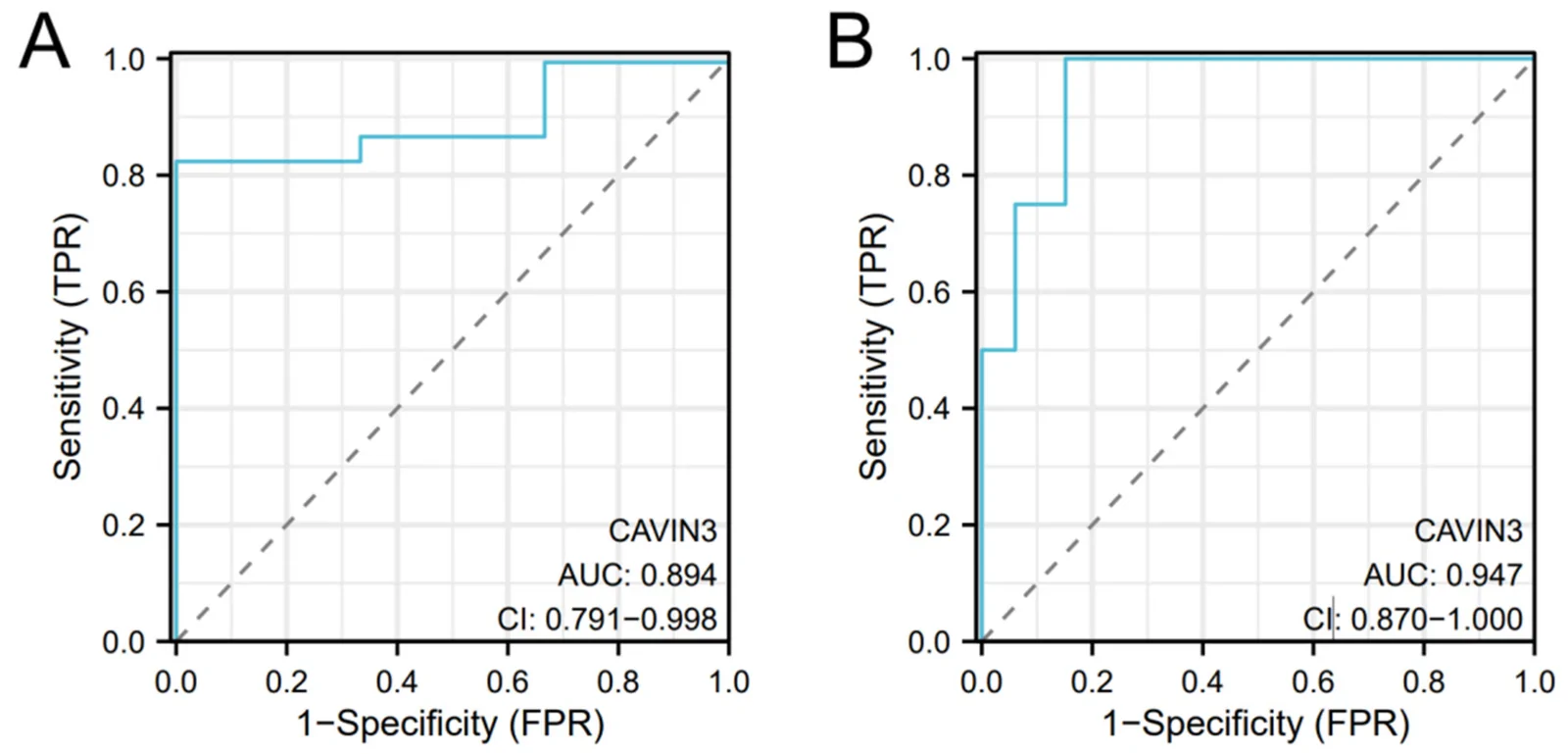
(**A**) Diagnostic ROC curve of CAVIN3 in cervical cancer based on TCGA database. (**B**) Diagnostic ROC curve of CAVIN3 in cervical cancer based on our center database.

To evaluate the diagnostic value of CAVIN3 in cervical cancer, we plotted ROC curves using TCGA RNA-seq data. The area-under-the-ROC curve (AUC) was 0.894, indicating favorable discriminative ability for sample stratification. Clinical cohort data confirmed this finding, with an AUC of 0.947 (Figure 7).

### 3.5. Functional Enrichment Analysis Results

**Figure 8:**
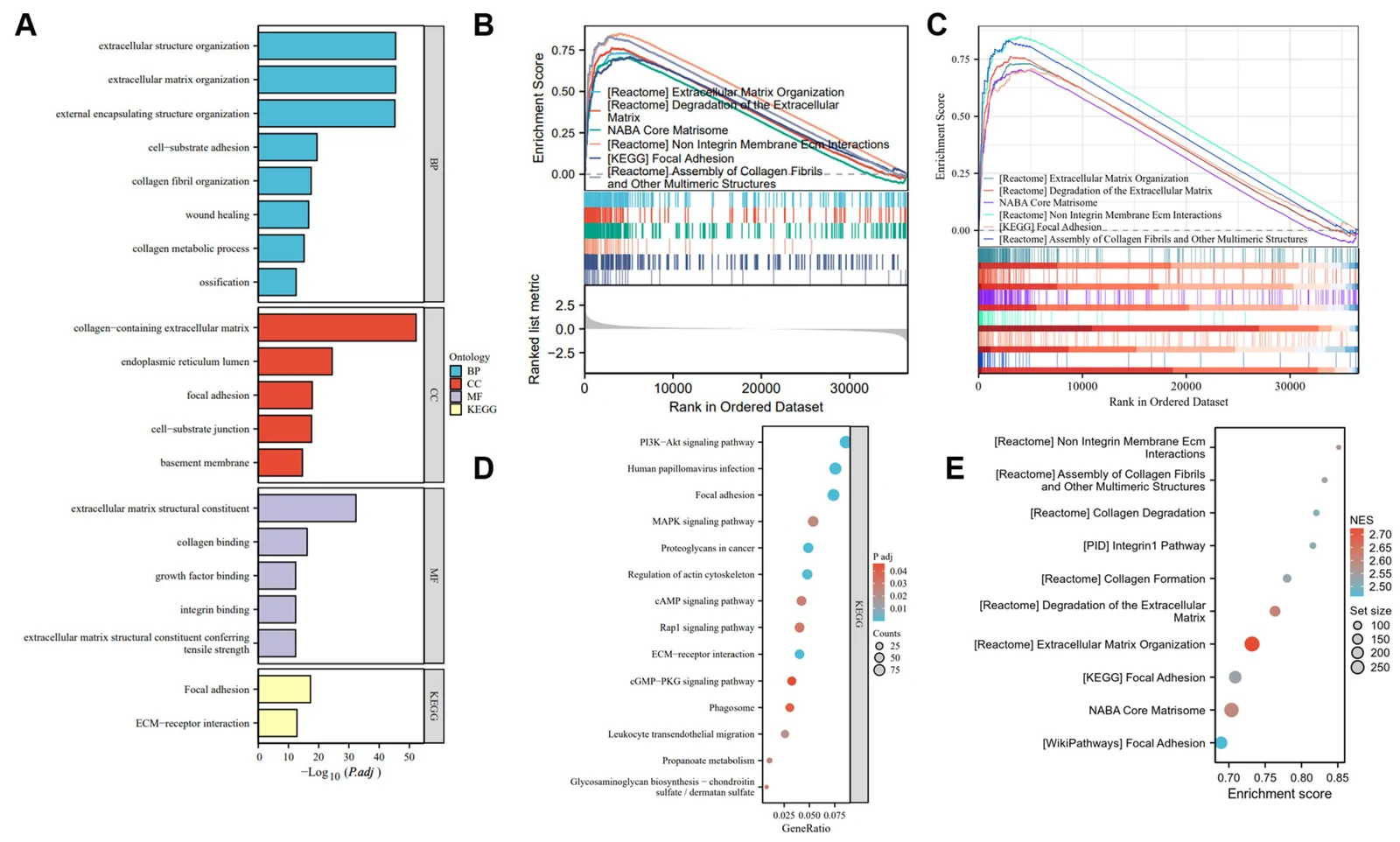
(**A**) Functional enrichment analysis of CAVIN3 in GO and KEGG pathways; (**B**,**C**): significantly enriched signaling pathways associated with CAVIN3 expression; (**D**,**E**) bubble plot visualization of pathway enrichment results.

To investigate the functions and signaling pathways of genes co-expressed with CAVIN3, we conducted GO and KEGG enrichment analyses. The results showed that the co-expressed genes in the high-CAVIN3-expression group were primarily enriched in processes including extracellular matrix production and degradation, extracellular matrix–cell membrane interactions, cell adhesion and migration, regulation of cell growth and differentiation, apoptosis, cellular homeostasis maintenance, and the integrin β1 signaling pathway (Figure 8).

## 4. Discussion

Cervical cancer arises from the malignant transformation of human cervical epithelial cells and is closely associated with persistent infection with high-risk human papillomavirus (HPV) [14,15]. Although the widespread implementation of vaccination and screening technologies has reduced the incidence of cervical cancer, it remains a significant burden to women’s health, with high incidence and mortality rates. Therefore, identifying reliable early diagnostic markers and new prognostic predictors is crucial for enhancing the survival outcomes of cervical cancer patients.

In recent years, caveolae—small, flask-shaped invaginations of the plasma membrane—have been investigated. They play key roles in processes like cell proliferation, apoptosis, migration, differentiation, angiogenesis, tumorigenesis, metastasis, endocytosis, and vesicular transport [16,17]. Caveolae components include caveolins, cavins, lipids, polymerases, and various ion channel proteins. Cavins are proteins associated with caveolae, among which is CAVIN3. The protein encoded by the CAVIN3 gene, also known as PRKCDBP, binds protein kinase C and delta [18,19].

This study systematically evaluated the expression characteristics, diagnostic value, and association with radiotherapy prognosis of CAVIN3 in cervical cancer. We found that the expression level of CAVIN3 in cervical cancer tissues was significantly lower than that in normal cervical tissues (TCGA cohort *p* = 0.019; our center cohort *p* = 0.002). CAVIN3 expression exhibited favorable sample discrimination, with AUC values of 0.894 in the TCGA cohort and 0.947 in our institutional cohort, reflecting its capacity to separate cervical cancer and non-malignant samples. This result is consistent with reports that the Cavin family (including CAVIN3, also known as PRKCDBP) plays a tumor-suppressive role in various malignant tumors [20]. Previous literature indicates that CAVIN3 exerts antitumor effects in breast cancer and lung cancer through mechanisms such as inhibiting the PI3K/AKT signaling pathway and facilitating DNA damage repair, while its downregulation is often associated with promoter hypermethylation-mediated silencing [21]. Notably, a systematic review [22] highlighted that the roles of Cavin family members (Cavin1–4) in tumorigenesis exhibit significant tissue specificity and context dependence, indicating that the same gene may have diametrically opposed functions in different tissue types or microenvironments. This functional diversity may arise from differences in Cavin complex assembly in various cellular environments and the divergence of downstream signaling pathways. But all patients in this study received radical or palliative radiotherapy and underwent no surgical intervention; consequently, post-resection pathological specimens were unavailable. CAVIN3 gene expression levels were assessed in biopsy tissues obtained for diagnostic purposes, without subsequent validation in independent diagnostic cohorts or postoperative specimens. Due to the inherent sampling limitations of biopsy tissue samples, further validation using postoperative pathological tissues is warranted to assess the clinical translational potential of CAVIN3.

Radiotherapy is a cornerstone of cancer treatment, inducing tumor cell apoptosis and necrosis through direct DNA damage (single-strand breaks [SSBs] and double-strand breaks [DSBs]) or indirectly via reactive oxygen species (ROS) and free radical formation [23]. Radiotherapy also enhances immune surveillance by promoting tumor-specific antigen production and cytotoxic T-cell activation, facilitating tumor clearance [24]. Additionally, it induces immunogenic cell death by releasing cytokines, inflammatory mediators, and other immune-related molecules [25]. Despite its efficacy, radioresistance remains a major challenge in cervical cancer treatment. Tumor response to radiotherapy depends on the “5 Rs”: DNA damage repair, cell cycle redistribution, repopulation, reoxygenation, and intrinsic radiosensitivity [26]. Understanding these mechanisms is critical for developing strategies to improve radiotherapy outcomes.

This study demonstrated a significant association between CAVIN3 expression levels and radiotherapy-related clinical outcomes in cervical cancer. Among patients receiving radiotherapy, the low CAVIN3 expression group showed a significantly higher objective response rate (88.2% vs. 56.5%, *p* = 0.006) and prolonged survival outcomes (median OS: not reached vs. 31.8 months, *p* = 0.043; median PFS in this cohort: 59.9 months vs. 9.8 months, *p* < 0.05). These observations reveal correlational links between CAVIN3 expression and both radiotherapy response and survival endpoints among cervical cancer patients. Given the limited sample size, further validation with a larger cohort is required. Mechanistically, our gene set enrichment analysis (GSEA) revealed that the pathways enriched in the high CAVIN3 expression group are predominantly associated with extracellular matrix (ECM) [27] formation and remodeling, ECM-cell membrane interactions, cell adhesion and migration, and the integrin β1 signaling pathway. The extracellular matrix is a dynamic three-dimensional network composed of macromolecules such as collagen, laminin, and fibronectin. It provides physical support to cells and participates in biological processes including cell adhesion, migration, and signal transduction, serving as a key component of the tumor microenvironment [28]. Its dynamic remodeling is closely associated with the development of radio-resistance. These pathways point to a more differentiated, matrix-adhesive phenotype, which may promote radio-resistance by enhancing cell survival signaling and DNA repair capacity. Recent studies [29] have found that CAVIN3 deficiency can downregulate Jagged1 (JAG1) expression by inhibiting ERK phosphorylation, further disrupting embryonic cell proliferation and vascular sprouting. The revealed CAVIN3-ERK-JAG1 signaling axis is universal: in cervical neoplasms, high CAVIN3 expression may maintain JAG1 expression through activation of ERK phosphorylation, thereby promoting abnormal angiogenesis and ECM remodeling, forming a microenvironment conducive to radiation resistance; whereas low CAVIN3 expression weakens this protective matrix feedback due to attenuated ERK signaling, thus enhancing radiosensitivity.

Furthermore, a prognostic model for cervical cancer based on PANoptosis-related genes has incorporated CAVIN3 as one of the risk genes [30]. This model demonstrates robust prognostic stratification ability in both TCGA and Gene Expression Omnibus (GEO) validations, and suggests that low-risk patients (i.e., those with lower CAVIN3 expression) exhibit stronger immune infiltration (such as NK cells and dendritic cells) and higher immune checkpoint expression, while high-risk patients (i.e., those with higher CAVIN3 expression) show features of ECM remodeling, PI3K-Akt activation, and immunosuppression. This supports our hypothesis from the perspective of the immune microenvironment: low CAVIN3 expression not only enhances the intrinsic radiosensitivity of tumor cells but may also synergistically enhance the effects of radiotherapy by promoting antitumor immune responses. Therefore, the expression status of CAVIN3 may serve as a potential biomarker for strategies combining radiotherapy and immunotherapy.

In summary, this study identifies the capacity of CAVIN3 expression to distinguish cervical cancer from non-malignant cervical tissues and confirms a statistically significant correlation between CAVIN3 levels and radiotherapy-related clinical outcomes as well as survival endpoints. Low CAVIN3 expression correlates with improved radiotherapy response and prolonged survival, and its underlying molecular mechanisms may involve the attenuation of ECM remodeling mediated by the ERK/JAG1 signaling pathway and the activation of an antitumor immune microenvironment.

## 5. Conclusions

CAVIN3 downregulation is a frequent event in cervical cancer and may contribute to tumor susceptibility. Paradoxically, within tumors, low expression of CAVIN3 correlates with improved radiotherapy response, likely stemming from the underlying phenotype characterized by poor differentiation and heightened radiosensitivity. These dual associations connect CAVIN3 expression to cervical cancer occurrence and interpatient differences in radiotherapy efficacy.

## Figures and Tables

**Table 1:** Baseline data of patients in our center.

Characteristics	Low CAVIN3, *n* = 56	High CAVIN3, *n* = 10
Age (years)		
<56	20 (35.7%)	1 (10%)
≥56	36 (64.3%)	9 (90%)
FIGO (2018)		
3	31 (55.4%)	3 (30%)
2	16 (28.6%)	5 (50%)
4	9 (16.1%)	2 (20%)
N stage		
0	36 (64.3%)	7 (70%)
1	20 (35.7%)	3 (30%)
Diameter, *n* (%)		
<4.8 cm	26 (46.4%)	3 (30%)
≥4.8 cm	30 (53.6%)	7 (70%)
Pathological type, *n* (%)		
Adenocarcinoma	9 (16.1%)	0 (0%)
Squamous cell carcinoma	47 (83.9%)	10 (100%)
ECOG score, *n* (%)		
ECOG 0	1 (1.8%)	0 (0%)
ECOG 1	55 (98.2%)	10 (100%)

**Table 2:** Prognosis-associated Cox proportional hazards regression analysis using TCGA database.

Characteristics	Total	Univariate Analysis		Multivariate Analysis	
		HR (95% CI)	*p*-Value	HR (95% CI)	*p*-Value
BMI	43				
1	13				
2	2	0.000 (0.000–Inf)	0.999		
3	28	1.777 (0.367–8.590)	0.475		
History of hormones	43				
1	15				
0	28	1.146 (0.285–4.609)	0.848		
CAVIN3	43				
L	23				
H	20	5.068 (1.049–24.487)	0.043	13.666 (1.473–126.764)	0.021
Number of productions	43				
1	25				
0	18	0.902 (0.241–3.379)	0.878		
Histology	43				
1	37				
2	6	2.210 (0.458–10.671)	0.323		
Age	43				
2	19				
1	24	0.666 (0.179–2.485)	0.546		
Histological grade	43				
G2	21				
G3	20	0.543 (0.136–2.171)	0.387		
G1	2	0.000 (0.000–Inf)	0.998		
HPV-positive	43				
0	40				
1	3	1.129 (0.140–9.128)	0.909		
Stage	43				
1	9				
3	12	2.153 (0.223–20.780)	0.507	1.153(0.117–11.392)	0.903
4	2	18.115 (1.542–212842)	0.021	45.655 (2.495–835.382)	0.010
2	20	1.314 (0.136–12.661)	0.813	0.681 (0.068–6.802)	0.743

**Table 3:** Prognosis-associated Cox proportional hazards regression analysis using our center database.

Characteristics	Total (N)	Univariate Analysis		Multivariate Analysis	
		Hazard Ratio (95% CI)	*p* Value	Hazard Ratio (95% CI)	*p* Value
Diameter	66				
<4.8 cm	29	Reference		Reference	
≥4.8 cm	37	3.004 (1.271–7.100)	0.012	2.943 (1.242–6.974)	0.014
Stage	66				
2	19	Reference			
3	36	1.986 (0.732–5.393)	0.178		
4	11	2.656 (0.807–8.746)	0.108		
N stage	66				
0	43	Reference			
1	23	1.148 (0.528–2.493)	0.728		
Age	66				
≤56	35	Reference			
>56	31	0.676 (0.317–1.444)	0.312		
CAVIN3	66				
L	56	Reference		Reference	
H	10	3.305 (1.388–7.871)	0.007	3.203 (1.334–7.688)	0.009

**Table 4:** Therapeutic efficacy evaluation summary in TCGA.

		Efficacy Evaluation		Total
		SD + PD	PR + CR	
Group	H	10	13	23
	L	4	30	34
	Total	14	43	57

**Table 5:** Cox proportional hazards regression analysis of CAVIN3 prognostic value with post-radiotherapy progression-free survival (PFS) as the endpoint in our center.

Characteristics	Total (N)	Univariate Analysis		Multivariate Analysis	
		Hazard Ratio (95% CI)	*p* Value	Hazard Ratio (95% CI)	*p* Value
Diameter	66				
1	37	Reference		Reference	
0	29	0.366 (0.174–0.771)	0.008	0.468 (0.207–1.056)	0.068
Stage	66				
4	11	Reference		Reference	
3	36	0.537 (0.234–1.230)	0.141	0.492 (0.187–1.295)	0.151
2	19	0.251 (0.086–0.733)	0.011	0.320 (0.084–1.226)	0.096
N stage	66				
0	43	Reference		Reference	
1	23	1.854 (0.936–3.675)	0.077	1.059 (0.450–2.493)	0.895
Age	66				
1	35	Reference			
0	31	0.799 (0.406–1.573)	0.516		
CAVIN3	66				
H	10	Reference		Reference	
L	56	0.417 (0.180–0.965)	0.041	0.373 (0.157–0.885)	0.025

## Data Availability

The 900th Hospital of PLA Joint Logistic Support Force (Fuzhou General Hospital of Nanjing Military Command, Fuzhou, China) datasets that support the findings of this study are available from the hospital, but restrictions apply to the availability of these data, which were used under licence for the current study and so are not publicly available. The data are, however, available upon request and with the permission of The 900th Hospital of PLA Joint Logistic Support Force (Fuzhou General Hospital of Nanjing Military Command, Fuzhou, China).
